# Scar Tissue Catheter Tip Occlusion From an Intrathecal Baclofen Delivery: A Case Report and Review of the Literature

**DOI:** 10.7759/cureus.70720

**Published:** 2024-10-02

**Authors:** James W Leiphart, Thaddeus J Leiphart

**Affiliations:** 1 Neurosurgery, University of Virginia School of Medicine, Falls Church, USA; 2 Neurosurgery, Inova Health System, Falls Church, USA

**Keywords:** catheter-tip granuloma, drug withdrawal, intrathecal baclofen therapy, intrathecal opioids, muscle spasticity

## Abstract

Intrathecal morphine is associated with the complication of catheter tip granuloma which causes symptoms of decreased pain control, increased required dose, and neurological deficit. Catheter tip granulomas from intrathecal baclofen are thought to never occur because of the mechanism by which intrathecal morphine causes granulomas. We present a case of an intrathecal baclofen induced scarring of a catheter tip with clinical characteristics similar to some symptoms of granuloma.

A 66-year-old woman with multiple sclerosis induced spasticity was partially controlled with intrathecal baclofen delivery at an extremely high dose of 1638 micrograms per day. She presented in the hospital with symptoms of withdrawal from intrathecal baclofen and required an emergency revision of her baclofen pump. Replacement of the catheter demonstrated complete occlusion of the catheter tip by scar tissue. Following surgery, her spasticity was well-controlled at the much lower dose of 200 micrograms per day.

Intrathecal baclofen delivery can cause catheter tip scarring which causes some symptoms similar to catheter tip granuloma. Early recognition of these signs of catheter tip occlusion could help prevent progression to withdrawal.

## Introduction

Delivery of intrathecal medications via intrathecal catheter and pump can be an effective therapy in select patient populations. Intrathecal baclofen is used for treatment of lower extremity spasticity from a variety of causes, and multiple different intrathecal medications are used for the treatment of chronic intractable pain. Intrathecal baclofen has demonstrated efficacy in the treatment of severe spasticity, with one study reporting 99.1% of patients continuing their therapy past the replacement of their intrathecal pump at the end of the pump battery life [1]. Intrathecal therapy is sometimes associated with complications when treating both spasticity [2-4] and pain [5]. The complication of catheter tip granuloma associated with intrathecal opiates for chronic intractable pain is especially concerning as it can lead to neurological deficit [6-11]. Very few cases of granulomas have been reported in patients with intrathecal baclofen pumps [12-15], and some have argued that intrathecal baclofen granulomas are impossible because it is the opiate drug that stimulates the inflammatory process causing the granuloma [16-18]. The typical intrathecal baclofen catheter complication is catheter blockage which has been reported without catheter location specified or in mid catheter [2-4]. Intrathecal baclofen withdrawal from catheter occlusion is typically sudden onset, and the withdrawal can lead to death [19]. Because of this, clinicians are vigilant for sudden onset withdrawal symptoms in intrathecal baclofen patients as a sign of pump or catheter failure. In contrast, catheter tip granulomas present with decreased pain control, increased requirement of medication and neurological deficit [7-9,11]. Because granulomas are rare or do not occur with intrathecal baclofen infusion, neurological deficit would not be expected, but any scarring or inflammatory reaction at the end of a catheter delivering baclofen could lead to a gradual reduction in symptom control and increase in required dose of baclofen. Because of this, distal catheter occlusion should be considered in intrathecal baclofen pump patients who experience gradual reduction in their symptom control and increased requirement of medication. In this case report, we discuss a case of distal catheter occlusion with scar tissue in an intrathecal baclofen patient that had gradually decreasing spasticity control and an increase in intrathecal baclofen requirement. The catheter tip occlusion was not considered until the patient went into sudden withdrawal because catheter tip pathology is not expected in intrathecal baclofen infusion.

## Case presentation

A 66-year-old woman with a past medical history of multiple sclerosis, Hirschsprung's disease, and neurogenic bladder requiring a suprapubic catheter presented with an intrathecal catheter and baclofen pump in place to treat her lower extremity spasticity. She has been non-ambulatory for 15 years. She has 3/5 strength in her right upper extremity, 1/5 strength in the left hand, and 0/5 strength in bilateral lower extremities. Her most recent pump replacement was three years prior, at which time her 40 cc SynchroMed II intrathecal baclofen pump was replaced with the same pump and the same 2000 microgram per milliliter concentration of baclofen. The intrathecal catheter with its tip at the T6 level was not replaced at that time. Her initial pump and catheter were placed six years prior to this pump revision with only one prior pump replacement.

She presented in the emergency department with signs and symptoms of baclofen withdrawal over the prior two weeks including worsening tremors, generalized itching, muscle spasms, generalized weakness, and hyperhidrosis. She was on an intrathecal baclofen dose of 1638 micrograms per day and was having her intrathecal pump refilled every five weeks. She reported that her pump was interrogated on the day of admission and found to have residual medication exceeding the expected amount of residual medication in the pump. An X-ray was performed which did not demonstrate a break or disconnection in the catheter. An MRI was not performed as there was no concern for a granuloma because she did not have a focal neurological deficit and it was a baclofen pump, not a morphine pump. She was admitted to the intensive care unit for observation. She was started on oral baclofen 20 mg every eight hours and valium as needed for breakthrough spasticity. She developed headache and diaphoresis with continued generalized itching and spasticity. Her oral baclofen was increased to 40 mg every eight hours, and she was taken urgently to surgery for an intrathecal pump exploration and replacement of the intrathecal catheter.

**Figure 1:**
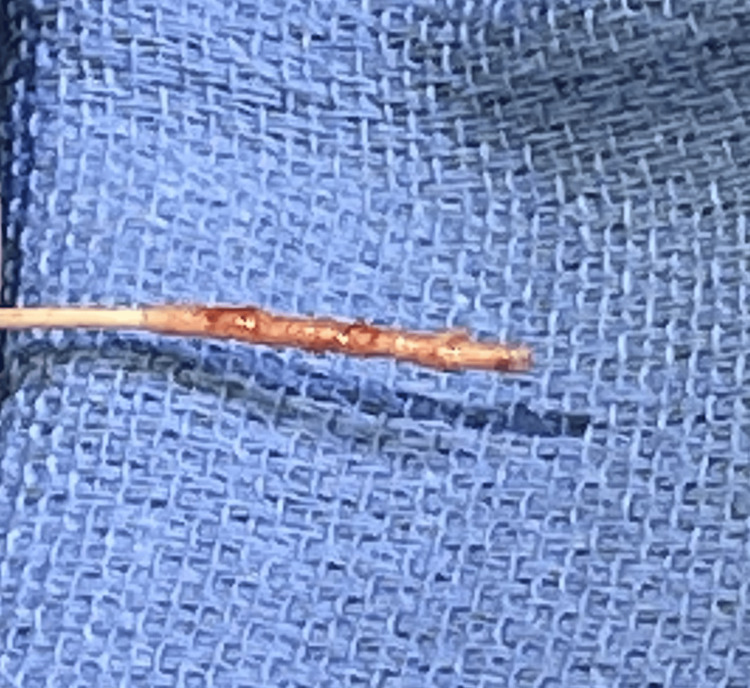
Photo after explant of scar tissue completely occluding the catheter tip from several years of intrathecal baclofen therapy

**Figure 2:**
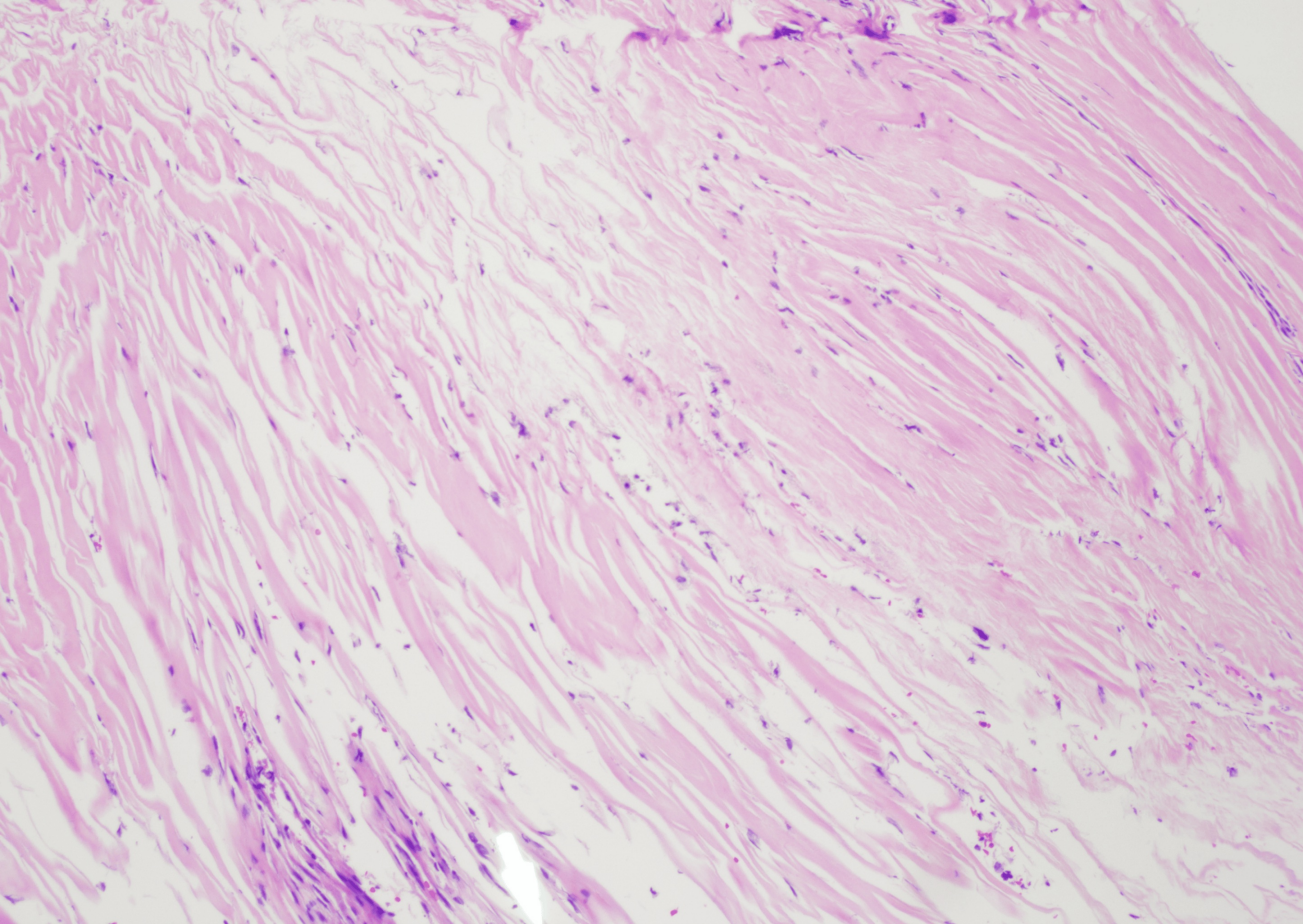
Microscopic pathology at 10x magnification of H&E stained scar tissue at the catheter tip demonstrates a lack of inflammatory cells. Inflammatory cells would be expected in a catheter tip granuloma H&E: Hematoxylin and eosin

During surgery, her intrathecal baclofen pump was tested and was found to be working effectively. Her intrathecal catheter had no flow. It was decided to replace the intrathecal catheter. Removal of the existing intrathecal catheter demonstrated significant initial resistance in pulling out the intrathecal portion of the catheter. Upon removal, fibrous tissue was observed at the end of the catheter blocking all the catheter holes (Figure 1). Pathological examination of the tissue occluding the end of the catheter did not demonstrate inflammatory cells consistent with a granuloma (Figure 2). The final diagnosis on the pathology report was all fibrous tissue with no evidence of granuloma. A new catheter was inserted, and the tip was advanced to the T6 level and attached to the functioning intrathecal pump. It was concluded that the blocked catheter was allowing no significant amount of baclofen through, so the patient was receiving no therapy. Because of this, the intrathecal baclofen dose was reduced to 200 micrograms per day to prevent sudden overdose. Over the next three days, she achieved good control of her spasticity and had complete resolution of her withdrawal symptoms. She was discharged to home on postoperative day three. 

## Discussion

This case report demonstrates an incidence of scarring at the tip of an intrathecal baclofen catheter which did not demonstrate the features of a granuloma on pathology but must represent an inflammatory process. The importance of this finding is that inflammation and scarring can occur at the catheter tip in intrathecal baclofen therapy demonstrating some of the classic signs of catheter tip granuloma including decreased control of symptoms and a progressively increased dose of intrathecal medication. Catheter tip granuloma formation is well-described in the literature, with estimates of occurrence rates varying widely from .49% to 43% of patients receiving intrathecal opiates [6,7,17]. The clinical characteristics of these granulomas are well-known including decreased pain control, progressively increasing dose necessary to relieve pain, and possibly neurological deficits related to spinal cord compression from the mass effect of the granuloma [7-11]. Diagnosis of intrathecal catheter tip granuloma usually only requires an MRI or CT myelogram for diagnosis as the space-occupying lesion at the tip of the catheter in patients receiving intrathecal opiates is characteristic [7,8,13]. The interventions for opiate-induced catheter tip granulomas are also well-described including repositioning the catheter tip out of the granuloma and decreasing the dose of opiate delivered [7,18], or in cases of progressive neurological deficit, open surgery for direct decompression [7,8,10,11]. The mechanism for intrathecal opiate catheter tip granuloma development is thought to be mast cell attraction from the vasculature of the dura and degranulation of the mast cells induced by the opiate medication present [7,16]. 

The proposed factors for intrathecal opiate-induced granulomas are not present in intrathecally delivered baclofen. Five catheter tip granulomas from intrathecal baclofen have been described in the literature [12,14,15]. It has been argued that the intrathecal baclofen catheter tip granulomas reported in the literature are misdiagnoses [16], citing the proposed mechanisms for intrathecal opiate granuloma formation [16,18]. A review of 1935 intrathecal baclofen pump complications reported no catheter tip granulomas [4]. A low incidence of catheter occlusion has been described for the delivery of intrathecal baclofen, but when described, it is not necessarily attributed to the catheter tip and not associated with an inflammatory process or a space-occupying lesion [2]. However, the clinical signs and symptoms of catheter occlusion are typically rapid in onset and are consistent with medication withdrawal including fever, spasticity increase, hypotension, changes in mental status, seizures, rhabdomyolysis, and disseminated intravascular coagulation (DIC), which can lead to death [19]. Because of this, clinicians who work with intrathecally delivered baclofen are vigilant of these signs of acute withdrawal. A more slow, indolent process characterized by the buildup of scar or inflammatory tissue as a result of inflammation may be missed. Without the mass effect of a granuloma, catheter tip occlusion via this slow process would be characterized by a slow loss of efficacy and a gradual increase in requited baclofen dose for therapeutic effect. This may be misinterpreted as a slow progression of the disease process rather than a slow catheter failure, and the true diagnosis may be missed because it is not as easily diagnosed as the catheter tip granuloma that can be seen on MRI. 

The present case study is an example of the need for increased awareness of the possibility that intrathecal baclofen delivery can lead to some inflammatory reaction that would cause slow catheter occlusion. In this case study, the patient’s intrathecal baclofen dose had been slowly increased over the years to a maximum dose of 1638 micrograms per day just prior to complete catheter occlusion and withdrawal symptoms. Given her diagnosis of multiple sclerosis, suspicion of slow catheter occlusion would have been generated at an increasing dose much lower than this if the possibility of intrathecal baclofen catheter tip inflammatory occlusion was widely known. Studies of effective intrathecal baclofen for the treatment of MS-associated spasticity have reported doses in the ranges of 26 to 725 micrograms per day, with mean values ranging from 163 to 211 micrograms per day [3,20]. Any intrathecally delivered baclofen therapy requiring greater doses than these should immediately prompt catheter evaluation which could include a fluoroscopic dye study or an empiric replacement of the intrathecal catheter if there is any question of its efficacy. An MRI alone is not enough as occlusion lacking granuloma formation would not be diagnosed by MRI. Early diagnosis and intervention is important as withdrawal from intrathecal baclofen is sudden, is difficult to manage, and can be lethal. 

## Conclusions

Although rare, this case study demonstrates a reactive intrathecal baclofen catheter tip occlusion. Awareness that this type of occlusion can occur and that it has some of the same signs and symptoms of catheter tip granuloma is important: it increases suspicion for these types of intrathecal baclofen therapy failures and allows elective intervention before a response to withdrawal is necessary. In cases like these, intervention would be surgical replacement of the catheter to continue intrathecal baclofen therapy, but the intrathecal baclofen dose would need to be reduced as the catheter tip occlusion would lead to higher doses of drug delivery to achieve the same effective dose. Early intervention is important as the resulting withdrawal can be life-threatening.
